# From the Petri dish to the patient’s bedside: interpreting *Trichosporon asahii* positive urine cultures in hospitalized patients

**DOI:** 10.3389/fimmu.2026.1866019

**Published:** 2026-06-29

**Authors:** Letícia Rebello Machado, Yasmim Passos Lima, André Netto Bastos, Victor Quinet de Andrade Bastos, Lucas Quinet de Andrade Bastos, Ricardo Villela Bastos, Vanessa Cordeiro Dias

**Affiliations:** 1 Biological Science, Federal University of Juiz de Fora (UFJF), Juiz de Fora, Brazil; 2 Department of Morphology, Federal University of Juiz de Fora (UFJF), Juiz de Fora, Brazil; 3 Cortes Villela Laboratory, Juiz de Fora, Brazil; 4 Department of Parasitology, Microbiology, and Immunology Federal University of Juiz de Fora (UFJF), Juiz de Fora, Brazil

**Keywords:** clinical outcome, hemoglobin, hemolysin, infection, leukocyte, treatment, *Trichosporon asahii*, urine

## Abstract

Urinary tract infections (UTIs) are common and traditionally linked to bacteria; however, yeasts— particularly *Trichosporon asahii* — have emerged as opportunistic pathogens in immunocompromised and hospitalized patients. The presence of fungi may represent either colonization or infection, and the understanding of immune bases can help in this differentiation. 19 *T. asahii* isolates from urine cultures of hospitalized patients were analyzed and subjected to antifungal susceptibility testing. Urine samples underwent routine analysis (appearance, protein, specific gravity, pH) and flow cytometry. Clinical and sociodemographic data were obtained from electronic medical records. Results: Of the 19 patients, 11 were catheterized and 8 non-catheterized, with a predominance of males aged ≥60 years. Most were admitted to the ICU (n=13), especially in the catheterized group. A high mortality rate was observed, particularly among catheterized patients (81.1%). Urinalysis demonstrated frequent abnormalities, such as proteinuria, hematuria, and leukocyturia, suggesting an active local immune response. In association with positive cultures for *T. asahii*, these findings reinforce the hypothesis of active urinary tract infection. The results highlight the relevance of accurate laboratory diagnosis of this pathogen and the implementation of appropriate therapeutic strategies based on antifungal susceptibility testing, clinical expertise, and pharmacokinetic and pharmacodynamic principles. These measures are essential to optimize clinical management, improve therapeutic outcomes, and reduce mortality associated with urinary tract infections caused by multidrug-resistant *T. asahii.*

## 1 Introduction

Urinary tract infections (UTIs) are among the most prevalent infectious conditions worldwide and represent a significant burden on global health systems (1). These infections affect individuals of all ages and both sexes; however, their incidence increases with age and exhibits a marked sex disparity, with women being approximately 3.6 times more likely to develop UTIs than men, and nearly half of all women experiencing at least one episode during their lifetime (2, 3). This higher susceptibility in women is largely attributed to anatomical and physiological factors, such as the shorter urethra and its proximity to the anus and vagina, which facilitate the ascending migration of uropathogens (4). Additionally, hormonal changes across the lifespan, particularly during menopause, further increase vulnerability to infection (5).

Clinically, UTIs may present either symptomatic or asymptomatic conditions and encompass a spectrum of manifestations depending on the site and severity of infection. Common symptoms include dysuria, urinary frequency, urgency, and hematuria, all of which can significantly impair quality of life. In more severe cases, particularly when infection progresses to the upper urinary tract or becomes systemic, patients may develop fever, hypotension, and altered mental status (6).

Historically, UTIs have been predominantly caused by bacterial pathogens, such as uropathogenic *Escherichia coli.* However, in recent decades, there has been a notable shift in the etiological profile, marked by an increasing contribution of fungal agents, particularly species of the genus *Candida* (7, 8). More recently, non-*Candida* yeasts have emerged as relevant opportunistic pathogens, especially among immunocompromised individuals and patients exposed to prolonged hospitalization, broad-spectrum antimicrobial therapy, and invasive procedures (9, 10).

In this context, *Trichosporon* spp., a genus of basidiomycetous yeast-like fungi widely distributed in the environment, has gained recognition as an opportunistic pathogen in humans, causing infections ranging from superficial lesions to life-threatening systemic diseases (11). Beyond their environmental ubiquity, these organisms may colonize the human host as part of the gastrointestinal, oral, respiratory, cutaneous, and vaginal microbiota (6). Among these species, *Trichosporon asahii* stands out as the most frequently implicated in invasive infections, such as bloodstream infections, peritonitis, and UTIs, predominantly affecting critically ill patients (10, 12–14).

Recent studies have reinforced the emerging role of *T. asahii* as an important urinary pathogen. A study conducted in İstanbul identified *T. asahii* as the most frequently isolated species of the genus in urinary tract infections, with a higher prevalence among patients admitted to intensive care units (82%), male individuals (68%), and elderly patients (66%) (15). In addition, a case of urinary tract infection caused by *T. asahii* has already been described in a young patient presenting urinary obstruction, nephrolithiasis, urinary catheterization, and multiple courses of antibiotic therapy (16). These findings highlight the clinical relevance of this yeast and reinforce its association with invasive procedures, prolonged hospitalization, and previous antimicrobial exposure.

The pathogenic potential of *T. asahii* is associated with a robust repertoire of virulence factors, including phospholipase activity, hemolysin production, and, most critically, biofilm formation (11–13).

The use of invasive medical devices, particularly indwelling urinary catheters, plays a central role in this pathogenesis by providing a surface for microbial adhesion. These biofilms create a protective niche that enhances microbial persistence and resistance to both the host’s immune response and antifungal agents (12, 17, 18). Consequently, catheter-associated urinary tract infections (CAUTIs) represent a significant proportion of healthcare-associated infections (HAIs) (19, 20).

The innate immune system constitutes the first line of biological defense, playing a decisive role in the early response against invaders. This mechanism is based on the detection of conserved molecular components shared by various microorganisms (21). In the case of fungal infections, essential structures of the cell wall — such as β-glucans, chitin, and mannans — serve as primary targets for recognition by innate immune cells (22, 23). Among the various cell types involved in this surveillance, macrophages and neutrophils perform crucial functions in the initial control of fungal load and the maintenance of tissue homeostasis (24).

The presence of fungi may represent either colonization or infection. Colonization refers to the presence of fungi without tissue invasion or disease, whereas infection involves tissue invasion, damage, potential systemic spread, and an associated inflammatory response (25). The same species can act as either a colonizer or a pathogen depending on factors such as the ecological niche, inoculum size, and host immune status. In invasive disease, the immune response is characterized by the recruitment of leukocytes, particularly neutrophils and macrophages, which aim to control fungal proliferation but may also contribute to tissue damage (26). This overlap can lead to misinterpretation: fungal infections may be mistaken for colonization and remain untreated, resulting in poor outcomes, while colonization may be misdiagnosed as infection, leading to unnecessary treatment (10). A better understanding of the underlying immunological mechanisms is essential to accurately distinguish between these two conditions.

Given the significant gaps in the management and treatment of individuals affected by infections caused by *Trichosporon*, there is a clear need for more precise and reliable diagnostic criteria. In this context, the present study aims to evaluate potential biomarkers, with an emphasis on leukocytes as indicators of the innate immune response, to differentiate infection from colonization by *T. asahii* in the urinary tract of hospitalized individuals. Additionally, it seeks to correlate these findings with clinical, sociodemographic, and microbiological aspects, thereby contributing to more accurate and evidence-based clinical decision-making.

## 2 Materials and methods

### 2.1 Ethical approval

The research was conducted with the consultation and consent of the participants, in compliance with the approved protocol by the Research Ethics Committee of UFJF (CAAE 18611019.6.0000.5147).

### 2.2 Study design and participants

This study adopted a descriptive, experimental, and cross-sectional design, focusing on 19 unique isolates of *T. asahii* recovered from urine cultures of hospitalized patients. The isolates were provided by a clinical microbiology laboratory located in Juiz de Fora, Minas Gerais, Brazil, and were collected between January 2021 and December 2023.

Eligible participants were those who had a positive urine culture for *T. asahii*, with no restrictions regarding age or sex.

The participating healthcare institution is a private hospital with approximately 160 beds, comprising specialized units, including adult and neonatal intensive care units (ICUs), coronary and neurological units, medical and surgical wards, as well as outpatient care services.

### 2.3 Review of clinical and sociodemographic data

Clinical and sociodemographic information was retrieved through a systematic review of electronic medical records of individuals with positive *T. asahii* isolates. The collected data included demographic characteristics (age, and sex), unit of origin, use of a urinary catheter, previous use of antibacterial agents, antifungal therapy following diagnosis, clinical outcome, reason for hospitalization, and outcome after antifungal therap. These data were compiled and organized using a standardized electronic spreadsheet.

### 2.4 Microbiological analysis

Urine samples were inoculated onto Sabouraud Dextrose agar (Neogen of Brazil, Lansing, USA) and incubated under aerobic conditions at 35 °C for up to 30 days. After this period, the test was considered negative in the absence of fungal growth. Positive cases for *T. asahii* were characterized by the development of beige to cream-colored, dry, cerebriform colonies.

The isolates were then subjected to biochemical and physiological identification using the automated Vitek 2^®^ system (bioMérieux, Marcy-l’Étoile, France), according to the manufacturer’s instructions. The reference strain *T. asahii* ATCC 90039 (American Type Culture Collection, Manassas, VA, USA) was used as the quality control standard, yielding a 99.9% identification match and thereby validating the identification process.

### 2.5 Visual evaluation and reflectance

Urine samples were subjected to visual inspection of appearance and classified according to their aspect as clear or turbid.

Protein, specific gravity, and pH were evaluated using the UC-3500 analyzer (Sysmex Corporation, Kobe, Japan) by reflectance.

### 2.6 Flow cytometry

Simultaneously, urine samples were analyzed by flow cytometry using the UF-5000 (Sysmex Corporation, Kobe, Japan) for the determination of epithelial cells, red blood cells, and leukocytes/pyocytes.

### 2.7 Antifungal susceptibility testing

The antifungal susceptibility profile of the *T. asahii* isolates was determined according to the guidelines established by the Clinical and Laboratory Standards Institute (27). Susceptibility testing was performed using the disk diffusion method with commercially prepared antifungal disks containing fluconazole (25 μg), voriconazole (1 μg), amphotericin B (20 μg), and caspofungin (5 μg), supplied by Liofilchem Diagnostics (Roseto degli Abruzzi, Italy).

The interpretive criteria for amphotericin B (20 μg) followed the parameters described by Menezes et al. (28), in which inhibition zones > 19 mm were considered susceptible, zones between 14 and 19 mm were classified as SDD, and zones < 14 mm were considered resistant. For fluconazole (25 μg), isolates presenting inhibition zones ≥ 19 mm were classified as susceptible, those with zones ranging from 15 to 18 mm as susceptible dose-dependent (SDD), and those with zones ≤ 14 mm as resistant, according to the criteria proposed by Pfaller et al. (29). Similarly, for voriconazole (1 μg), inhibition zones ≥ 17 mm were considered susceptible, zones between 14 and 16 mm were classified as SDD, and zones ≤ 13 mm were interpreted as resistant (30).

### 2.8 Statistical analysis

Descriptive statistical analyses were performed, including absolute frequencies, percentages, ranges, and mean age values of the patients. Categorical variables were compared using Fisher’s exact test due to the small sample size and low expected frequencies. Statistical significance was considered at *p* < 0.05.

## 3 Results

A total of 19 individuals with positive urine culture for *T. asahii* were included, of whom 11 were catheterized and 8 non-catheterized. In both groups, there was a predominance of male individuals and aged ≥60 years. Among catheterized individuals, acute renal failure was the most frequent reason for hospitalization, whereas in non-catheterized individuals, a more heterogeneous distribution of underlying conditions was observed, including cancer and other comorbidities (**Table 1**).

**Table 1:** Clinical and sociodemographic aspects of individuals with a positive urine culture for *T. asahii* (n=19).

Clinical and epidemiological parameters	Catheterized individuals (n= 11)	Non catheterized individuals (n=8)	*p*-value
Gender: n (%)			0.603
Female	3 (27.3)	1 (12.5)	
Male	8 (72.7)	7 (87.5)	
Age: n (%)			0.773
≤ 12 years	1 (9.1)	0 (0.0)	
13–59 years	2 (18.2)	3 (37.5)	
≥ 60 years	8 (72.7)	5 (62.5)	
Reason for hospitalization: n (%)			0.205
Acute Renal Failure	4 (36.4)	1 (12.5)	
Cancer	0 (0.0)	2 (25.0)	
Hemorrhage	1 (9.1)	0 (0.0)	
Heart failure	0 (0.0)	1 (12.5)	
Liver failure	1 (9.1)	0 (0.0)	
Osteomyelitis	0 (0.0)	1 (12.5)	
Pressure ulcer	2 (18.2)	1 (12.5)	
Septicemia	0 (0.0)	1 (12.5)	
Respiratory Failure	1 (9.1)	0 (0.0)	
Respiratory Infection	2 (18.2)	0 (0.0)	
Collection unit: n (%)			0.603
ICU	9 (81.1)	4 (50.0)	
Inpatient unit	2 (18.2)	4 (50.0)	
Surgical center	0 (0.0)	0 (0.0)	
Previous use of antibacterial agents			0.660
No	0 (0.0)	0 (0.0)	
Yes	11 (100)	8 (100)	
Yes - Carbapenems	10 (90.9)	7 (87.5)	
Yes - Polymyxins	6 (54.5)	2 (25.0)	
Yes - Aminoglycosides	5 (45.5)	5 (62.5)	
Yes - Penicillins (± β-lactamase inhibitor)	5 (45.5)	5 (62.5)	
Yes - Glycopeptides	4 (36.4)	4 (50.0)	
Yes - Cephalosporins	4 (36.4)	2 (25.0)	
Yes - Oxazolidinones	2 (18.2)	2 (25.0)	
Yes - Fluoroquinolones	3 (27.3)	0 (0.0)	
Yes - Macrolides	1 (9.1)	1 (12.5)	
Yes - Antifungals	0 (0.0)	2 (25.0)	
Yes - Antivirals	1 (9.1)	1 (12.5)	
Yes - Tetracyclines	1 (9.1)	0 (0.0)	
Yes - Nitroimidazoles	1 (9.1)	0 (0.0)	
Clinical outcome:			0.603
Death	9 (81.1)	5 (62.5)	
Hospital discharge	2 (18.2)	3 (37.5)	
Use of antifungals after diagnosis:			0.319
No	2 (18.2)	4 (50.0)	
Yes	9 (81.1)	4 (50.0)	
Yes - Combination therapy	4 (36.4)	1 (12.5)	
Yes - Fluconazole	8 (72.7)	1 (12.5)	
Yes - Itraconazole	0 (0.0)	3 (37.5)	
Yes - Nystatin	2 (18.2)	0 (0.0)	

Clinical and sociodemographic aspects of catheterized and non-catheterized individuals with urinary isolation of *T. asahii*. Data are presented as absolute frequency and percentage [n (%)]. Comparisons between groups were performed using Fisher’s exact test, and *p*-values < 0.05 were considered statistically significant.

Most individuals were admitted to the ICU (n=13), particularly in the catheterized group. All individuals had a history of exposure to antibacterial agents, with a high prevalence of broad-spectrum agents in both groups. Carbapenems were the most frequently used class among patients with catheters (90.9%) and without catheters (87.5%). Notably, polymyxins were administered more frequently in patients with catheters (54.5% vs. 25.0%), suggesting greater concern with infections caused by multidrug-resistant Gram-negative bacteria in this group (**Table 1**).

Regarding clinical outcomes, a high mortality rate was observed (n=14), especially among catheterized individuals (81.1%). Antifungal therapy was administered in most cases (n=13), with fluconazole being the most used drug in catheterized individuals, while itraconazole was more frequently used in non-catheterized individuals (**Table 1**).

**Figure 1:**
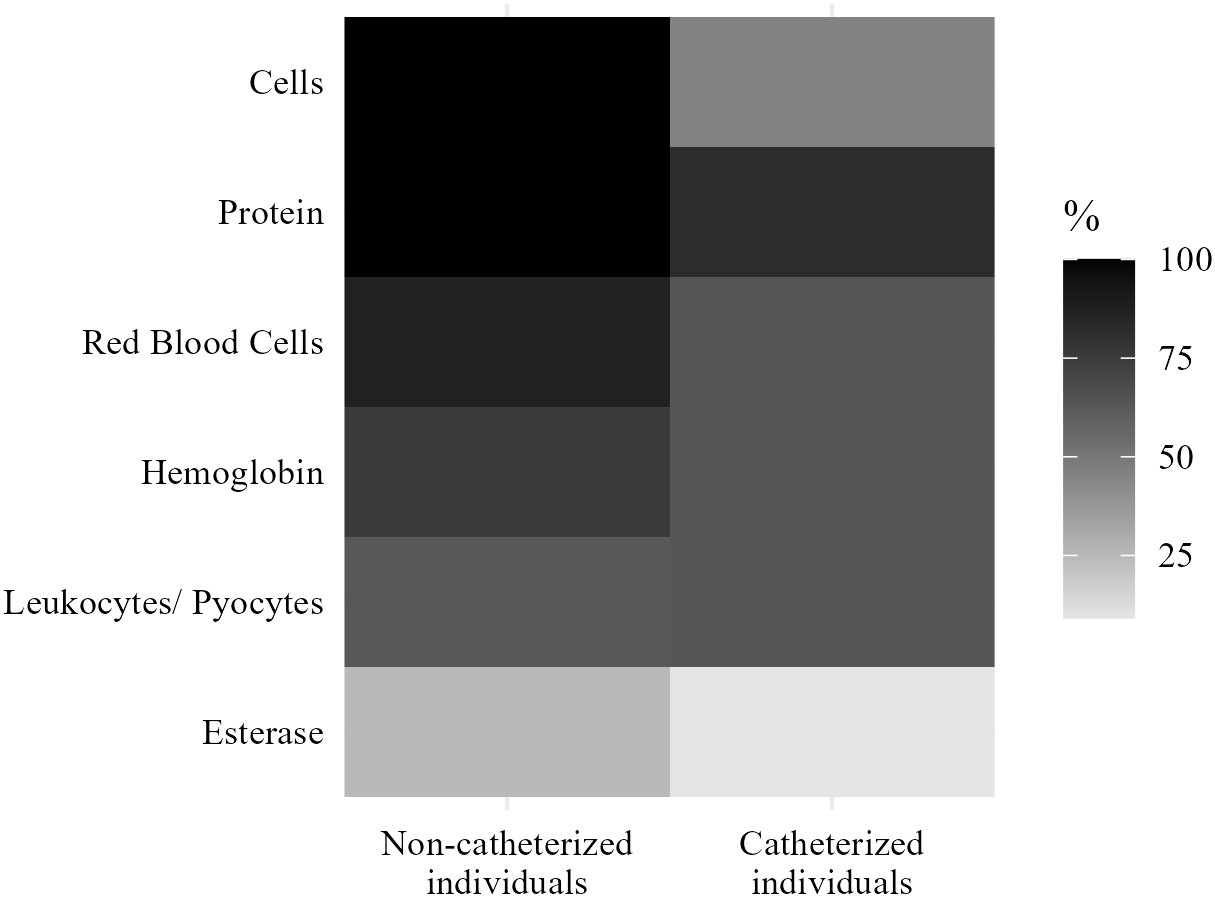
The following abnormal urinary elements were evaluated in catheterized and non-catheterized individuals: Cells (presence of cells in urine), Protein (presence of protein in urine), Red Blood Cells (red blood cells in urine), Hemoglobin (hemoglobin in urine), Leukocytes (leukocytes in urine), and Esterase (leukocyte esterase). Darker tones indicate elements classified as abnormal in the analysis, whereas lighter gray tones indicate elements classified as normal.

All samples presented two or more altered parameters in the urinalysis. Among individuals without a catheter, the main abnormalities observed were the presence of cells, proteins, and red blood cells at levels outside the normal range. In contrast, among individuals with a catheter, the primary alteration was elevated to urinary protein levels, followed by the presence of red blood cells, hemoglobin, and leukocytes/pyocytes (**Figure 1**).

Three samples (15.8%) showed abnormal values for both leukocyte count and esterase, while 9 samples (47.4%) presented alterations only in leukocyte levels, with esterase remaining within the reference range (**Figure 1**). Additionally, 8 samples (42.1%) exhibited concurrent abnormalities in protein, red blood cells, hemoglobin, and leukocyte levels (**Figure 1**).

**Figure 2:**
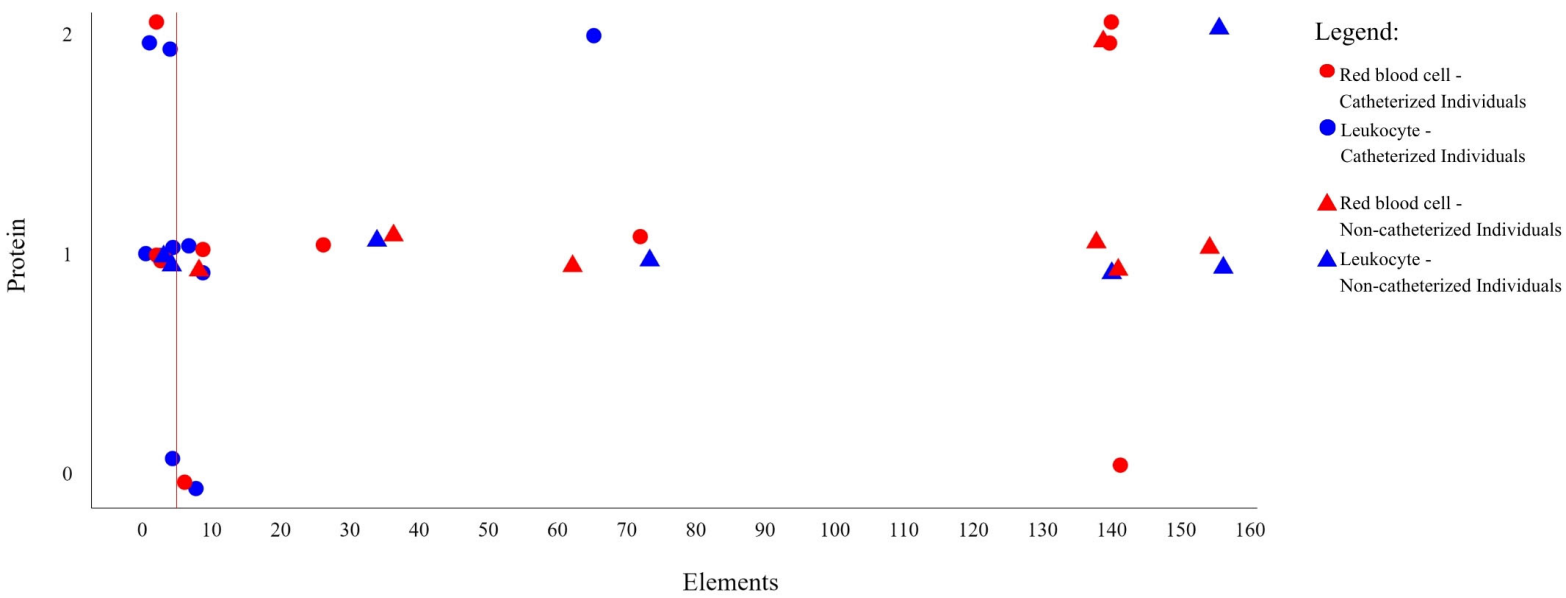
The scatter plot shows the relationship between protein and red blood cells (red) and between protein and leukocytes (blue) in the catheterized group (circles) and the non-catheterized group (triangles). The red line indicates the upper limit of normality for the test (maximum of 5 elements in urine).

The relationship between protein vs. red blood cells (RBC) in urine and protein vs. pyoocytes in urine (pyuria) was highly heterogeneous in both groups (catheterized and non-catheterized). Most samples (78.9%) were outside the reference threshold for RBC and pyoocytes. Additionally, 53.3% of these abnormal findings occurred simultaneously with the presence of proteins at the first level, while only 13.3% of the abnormalities occurred in the absence of protein (**Figure 2**).

**Figure 3:**
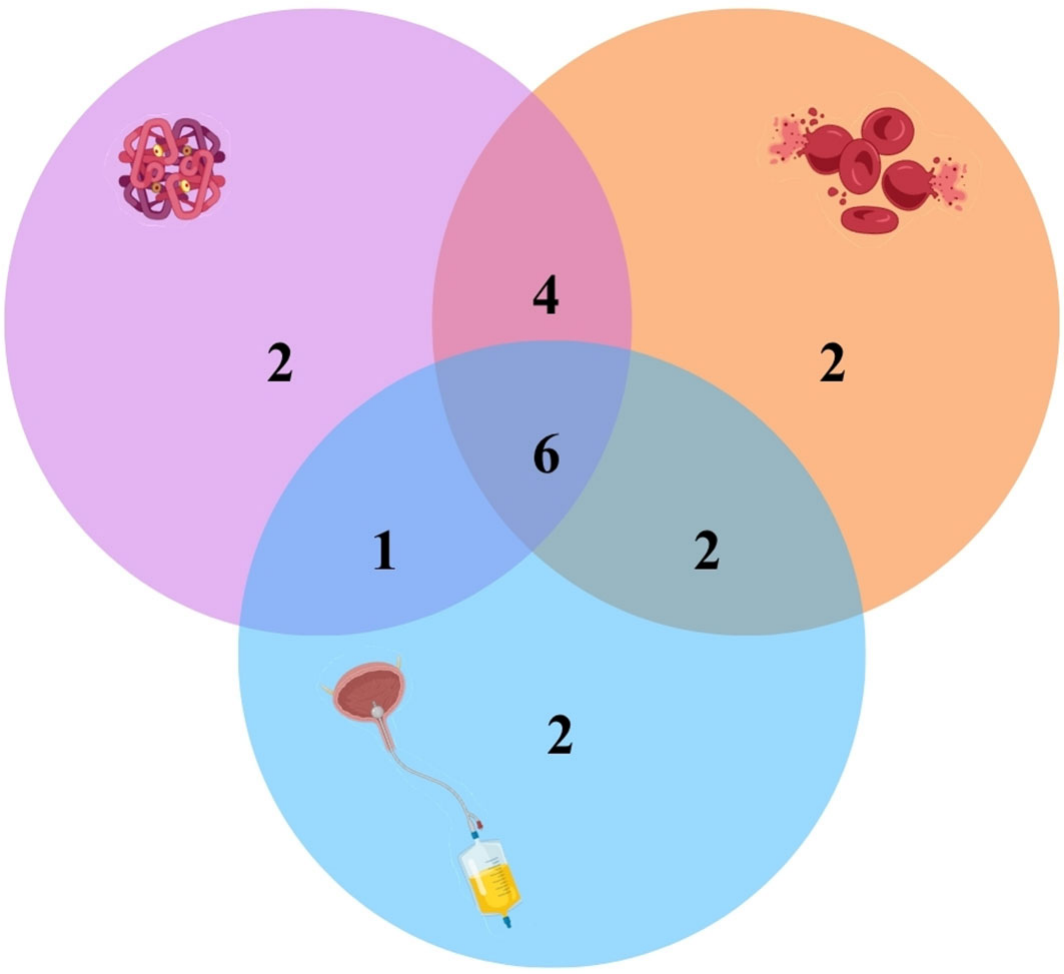
Venn diagram illustrating the distribution and overlap of hemoglobinuria (pink), hemosiderin (orange) and catheterization (blue) status. Numbers within each section represent the number of individuals presenting each condition alone or in combination with others.

The Venn diagram (**Figure 3**) illustrated the distribution and overlap among hemoglobin, protein, and catheterization. Four individuals exhibited an overlap between hemoglobin and protein, while a higher number of cases (n=6) demonstrated a correlation between urinary catheter use, proteinuria, and hemoglobinuria. Two patients without catheter use and with positive cultures for *T. asahii* showed no alterations in urinary protein or hemoglobin.

Regarding urinary characteristics, most samples were turbid (89.5%), whereas a smaller proportion appeared clear (10.5%). pH values below 7 were more frequent, occurring in 57.9% of cases. Urinary density was predominantly normal, accounting for 68.4% of the analyzed samples (**Table 2**).

**Table 2:** Integrated analysis of urinary findings in hospitalized individuals with positive culture for *T. asahii*.

Urine aspect	
Appearance: n (%)	
Clear	2 (10.5)
Turbid	17 (89.5)
pH: n (%)	
< 7	17 (89.4)
7	1 (5.3)
≥ 8	1 (5.3)
Density: n (%)	
< 1010	5 (26.3)
1010 - 1025	13 (68.4)
> 1025	1 (5.3)
Outcome after antifungal therapy: n (%)	
Death	10 (71.4)
Discharge	4 (28.6)

Urinary characteristics and clinical outcome after antifungal therapy in individuals with urinary isolation of *Trichosporon asahii*. Data are presented as absolute frequency and percentage [n (%)].

Among non-catheterized individuals (n = 8), all isolates were susceptible to fluconazole and voriconazole, while six (75%) isolates were susceptible to amphotericin B. Similarly, among catheterized individuals (n = 11), all isolates showed susceptibility to fluconazole and voriconazole, whereas nine (81.8%) isolates were susceptible to amphotericin B.

## 4 Discussion

The present findings delineate a severe clinical profile associated with urinary tract infections (UTIs) caused by *T. asahii*, predominantly affecting elderly male individuals regardless of catheter use. This is consistent with previous reports describing this species as the most clinically relevant within the genus, frequently associated with hospitalized and immunocompromised patients (11, 14).

Urinary catheterization is a well-established risk factor for fungal UTIs due to its role in facilitating microbial adhesion and biofilm formation (12, 17, 18). At the same time, catheter use may act as an important confounding factor, as mechanical irritation of the urinary epithelium can induce local inflammation and microtrauma, leading to hematuria and the presence of hemoglobin degradation products even in the absence of active infection. Consequently, urinary findings such as hemoglobinuria, turbidity, and physicochemical alterations may reflect not only infection-related processes but also catheter-associated mechanical effects (7, 19). This dual role of catheterization highlights the complexity of distinguishing infection from colonization and underscores the need to interpret urinalysis results in conjunction with microbiological and clinical data.

In this study, catheterized individuals exhibited indicators of greater clinical severity, including a higher frequency of acute renal failure as the primary cause of hospitalization and a predominance of intensive care unit (ICU) admissions. These findings suggest that critical illness and organ dysfunction may play a central role in susceptibility to infection and adverse outcomes (20, 28). In contrast, non-catheterized individuals presented a more heterogeneous distribution of underlying conditions, including malignancies and other comorbidities, indicating that host-related factors beyond medical device use also contribute to infection risk (13, 31). However, the interpretation of these clinical and laboratory findings requires careful consideration of urinary catheterization itself, which may influence both the risk of fungal infection and the urinary abnormalities observed.

Exposure to antibacterial agents, such as carbapenems (90.9%) and polymyxins (54.5%) among catheterized patients, suggests an association with multidrug-resistant Gram-negative bacterial infections. This reinforces the clinical complexity of this group and indicates a possible impact on their microbiota (32–34).

Notably, the high overall mortality rate (73.4%), particularly among catheterized patients (81.8%), supports the hypothesis that the interplay between invasive devices and prior antimicrobial exposure contributes substantially to poor clinical outcomes. Urinary catheterization is a well-known risk factor for biofilm formation and persistent colonization by opportunistic fungi, including *T. asahii*, which may hinder antifungal penetration and therapeutic efficacy (11–13).

Following laboratory confirmation by culture and urinalysis, 68.4% of patients were treated with antifungal agents, reflecting the clinical relevance attributed to these findings by the attending physicians. However, despite treatment, a substantial number of patients progressed to death, indicating that antifungal therapy alone may be insufficient to reverse outcomes in critically ill individuals. This may reflect both the patients’ prior clinical status and the *in vivo* antifungal efficacy against *Trichosporon* spp. Consistent with this interpretation, previous studies reported high mortality rates associated with *Trichosporon* infections, especially in cases involving delayed initiation of appropriate therapy or the use of agents with limited activity, such as echinocandins (11, 13, 35, 36). Collectively, these findings emphasize the importance of early diagnosis, rigorous risk factor evaluation, and timely initiation of appropriate antifungal therapy to mitigate the high mortality associated with *T. asahii* UTIs (13, 14, 37).

The isolation of *T. asahii* in urine culture alone does not justify antifungal intervention, and its clinical significance should be interpreted in conjunction with laboratory and clinical findings. In the present study, all analyzed samples exhibited two or more abnormalities in urinalysis parameters, and positive cultures were frequently associated with an inflammatory urinary profile characterized by leukocyturia, hematuria, and proteinuria. Furthermore, three samples presenting leukocyturia also exhibited increased leukocyte esterase levels. As leukocyte esterase is released during leukocyte activation within the urinary tract, its presence may indicate an ongoing inflammatory process (38, 39).

Collectively, these findings suggest an association between positive *T. asahii* cultures and urinary inflammatory abnormalities, supporting a potentially biologically relevant host–microorganism interaction. The concurrent occurrence of multiple urinary abnormalities may be compatible with an inflammatory urinary environment and could indicate the possibility of infection. However, the present data do not allow a definitive distinction between active infection and colonization by *T. asahii.* This limitation is particularly important given the presence of potential confounding factors, including urinary catheterization, critical illness, renal dysfunction, and other conditions capable of inducing urinary tract inflammation independently of fungal infection.

Fungal pathogens are known to activate host immune responses during infection, particularly through innate immune pathways (22, 23, 26). In the present study, an interrelationship was observed among hemoglobinuria, proteinuria, and urinary catheter use in individuals with positive *T. asahii* cultures, with the concurrent presence of all three conditions representing the most frequent pattern (n = 6). Additionally, leukocyturia was detected in 63.1% of samples, while 42.1% of cases exhibited simultaneous alterations in red blood cells, hemoglobin, protein, and leukocyte levels, suggesting the presence of an inflammatory urinary milieu. Nevertheless, the parameters evaluated in this study represent indirect markers of inflammation and do not provide direct evidence of innate immune pathway activation. Therefore, it is not possible to conclude that the observed inflammatory profile resulted specifically from host immune responses triggered by *T. asahii*. Rather, the findings support the hypothesis that positive cultures for this species are frequently associated with urinary inflammatory abnormalities, warranting further investigation. Future prospective studies integrating clinical, microbiological, and immunological data will be necessary to determine whether specific inflammatory urinary profiles can help differentiate colonization from infection in patients with positive *T. asahii* urine cultures.

The high frequency of turbid samples is consistent with the presence of cellular debris, microorganisms, and inflammatory components (40–42). The predominance of acidic pH may reflect host metabolic conditions or microbial activity, although *Trichosporon* spp. can tolerate a wide pH range (43, 44). Urinary density (specific gravity) remained within normal ranges in most cases (n=13/68.4%), suggesting preserved renal concentration capacity (40, 45).

This study has limitations that should be considered when interpreting the findings. The relatively small sample size limits the statistical power and generalizability of the results. Additionally, as a single-center study conducted in a tertiary-care hospital, the findings may not be representative of other healthcare settings or patient populations. The retrospective observational design also precludes the establishment of causal relationships between *T. asahii* isolation and the urinary abnormalities observed. Furthermore, although positive urine cultures were frequently associated with inflammatory urinalysis findings, the available data do not allow a definitive distinction between colonization and active infection. Therefore, the results should be interpreted as demonstrating an association rather than causation.

## 5 Conclusion

In conclusion, the detection of *T. asahii* in urine was frequently associated with urinalysis abnormalities, particularly leukocyturia, hematuria, and proteinuria, suggesting an inflammatory urinary environment and a potentially relevant host–pathogen interaction. Although routine urinalysis alone cannot distinguish fungal from bacterial etiologies, its integration with microbiological culture enhances diagnostic accuracy. These findings highlight the importance of combining laboratory and microbiological data for the identification of urinary fungal infections, especially in hospitalized and high-risk patients. Early recognition of *T. asahii* may support timely therapeutic decisions, reduce diagnostic delays, and contribute to improved patient management and clinical outcomes.

## Data Availability

Requests to access the datasets should be directed to vanessa cordeiro dias, vanessa.dias@ufjf.br.
